# Biomechanical analysis of maxillary posterior three unit bridge supported misial straight implant and distal tilted implant

**DOI:** 10.3389/fbioe.2025.1546656

**Published:** 2025-02-25

**Authors:** Guanqi Liu, Shudan Deng, Xiaoyan Chen, Jiahui Lin, Runheng Liu

**Affiliations:** Hospital of Stomatology, Guanghua School of Stomatology, Sun Yat-Sen University and Guangdong Provincial Clinical Research Center of Oral Diseases, Guangzhou, China

**Keywords:** dental implant, biomechanics, finite element analysis, maxillary sinus, implant angulation

## Abstract

**Purpose:**

This study aims to investigate the stress distribution in bone tissue, implant, abutment, screw, and bridge restoration when the mesial implant is placed axially and the distal implant is inserted at varying angles in the posterior maxillary region with free-end partial dentition defects, using three-dimensional finite element analysis.

**Materials and methods:**

Cone-beam computed-tomography were utilized to create 3D reconstruction models of the maxilla. Stereolithography data of dental implants and accessories were used to design a three-unit full zirconia bridge for the maxillary model. The 3D models were imported into ANSYS Workbench 23.0 software for mesh generation and material property definition. Five different distal implant implantation directions were designed: Inner Tilting 30° group, Inner Tilting 17° group, Parallel group, External Tilting 17° group, and External Tilting 30° group. The models consisted of cortical bone, trabecular bone, implants, abutments, central screws, prosthesis screws, and prostheses. Material properties were assumed to be isotropic, homogeneous, and linearly elastic. The maxillary models were subjected to strict fixation restrictions, and the implants were considered fully osseointegrated. Two loading types were set in ANSYS Workbench 23.0: a vertical load of 300N and a lateral load of 300N at a 45°angle to the implant.

**Results:**

Under vertical loading, the parallel group exhibited the lowest maximum stress across all implants, crowns, abutments and screws. Greater tilt angles increased abutment stress, with the external tilting 30° group reaching 1,426 MPa (close to titanium alloy’s yield strength). Smaller angles of both external tilting and inner tilting shifted stress to implants from abutment and screw. During lateral loading, the external tilting 30° group showed catastrophic stress escalation (abutment: 8,612 MPa), exceeding titanium’s yield limit. Bone stress remained physiological except for the internal tilting 30° group under lateral loading (142 MPa).

**Conclusion:**

The parallel group demonstrated the least stress accumulation in all components and bone tissues. Internal tilting of the distal implant is biomechanically preferable to external tilting, and a smaller tilt angle is recommended when external tilting is necessary. This study provides valuable reference data for optimizing implant angulation in patients with the loss of three posterior maxillary teeth, potentially reducing long-term complications associated with implant-fixed bridges.

## Graphical Abstract

**Figure:**
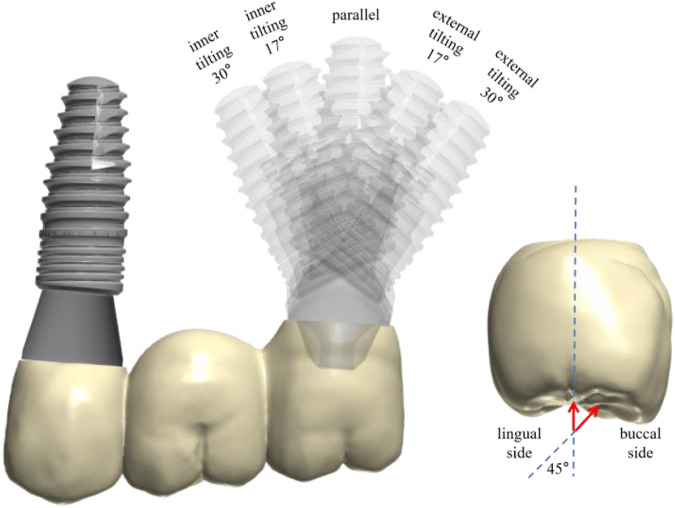
Graphical abstract illustrates the specific groupings and loading patterns in this study. Highlights: the parallel group demonstrated the least stress accumulation in all components and bone tissues. Internal tilting of the distal implant is biomechanically preferable to external tilting, and a smaller tilt angle is recommended when external tilting is necessary.

## Introduction

Patients with tooth loss in the posterior maxillary region may have insufficient remaining vertical bone volume in the posterior maxillary region due to pathological resorption, maxillary sinus pneumatization, and congenitally low positions of the maxillary sinus floor. Before implant surgery, using vertical bone augmentation methods to address the issue of insufficient vertical bone height in the posterior maxillary region has a substantial amount of evidence-based medical evidence and is a reliable clinical treatment plan (Lyu et al., 2023). Currently, in maxillary sinus elevation procedures, the external elevation technique through the lateral wall of the maxillary sinus and the internal elevation technique through the alveolar ridge crest of the edentulous area are the most commonly used means of sinus floor elevation (Gao et al., 2023; Alsharekh et al., 2024).

With the increasing emphasis on minimally invasive treatment, some scholars have proposed that the use of tilted implantation when bone volume is insufficient (Gümrükçü and Korkmaz, 2018). Tilted implantation involves placing the implant at an angle to the axial direction when there is insufficient residual alveolar bone height. This approach aims to avoid damaging important anatomical structures during implant surgery, such as the maxillary sinus, inferior alveolar nerve, nasal floor, and other key anatomical features. It also maximizes the use of the remaining bone volume and avoids extensive bone augmentation (Zhuang et al., 2024; Yu et al., 2022). Compared to maxillary sinus elevation procedures, tilted implantation has lower technical difficulty, reduces postoperative reactions in patients, has the same treatment period as conventional implants, and lower treatment costs. To date, tilted implantation has been primarily applied to treat edentulous patients using the All-on-4 technique (Liu et al., 2019; Ozan and Kurtulmus-Yilmaz, 2018; Ibrahim et al., 2024).

In recent years, with the widespread application of the All-on-4 technique, tilted implantation has been proven to be a reliable implant technique. The two distal implants in the maxilla are implanted obliquely along the anterior wall of the maxillary sinus, meaning the apices of the tilted implants are mesially, and the necks of the tilted implants are distally. Some clinicians also apply the tilted implant technique to the distal position of the maxillary sinus, which is the pterygoid plate-maxillary tuberosity area, to avoid complex maxillary sinus floor elevation procedures while achieving immediate repair in some cases. Currently, studies on the All-on-4 technique and pterygoid maxillary implant techniques are mostly applied in the implant treatment of edentulous maxillae, serving as free-end auxiliary support.

In clinical practice, free-end partial dentition defects in the posterior maxillary region (second premolar, first molar, and second molar all missing) are quite common. Due to treatment cost considerations, two implants are often inserted and a three-unit bridge restoration is applied. The conventional choice is to insert two parallel implants, however some doctors use the aforementioned tilted implant technique to avoid the maxillary sinus, but there are relatively few reports on biomechanical analysis. Finite element analysis (FEA) is a method that uses mathematical simulation to deduce the stress distribution and deformation of any given geometric shape structure in a real system, and can be widely used to simulate clinical situations and study the biomechanical behavior and mechanical properties of dental materials.

Therefore, the purpose of this study is to investigate the stress distribution and deformation of bone tissue, implants, abutments, screws, and restorations when the mesial implant is implanted axially and the distal implant is implanted at different angles in the posterior maxillary region with free-end partial dental defects, using three-dimensional finite element technology, to provide experimental evidence for improving the design of implant repair in the posterior maxillary region.

The main novelty of this research lies in the detailed biomechanical analysis of different implant angulations in the posterior maxillary region with free-end partial dentition defects. This study addresses the main question by investigating the stress distribution in bone tissue, implants, abutments, screws, and restorations when the mesial implant is implanted axially and the distal implant is implanted at different angles. By providing comprehensive data on stress distribution, this research offers valuable insights for optimizing implant design and reducing long-term complications associated with implant-fixed bridges in such clinical scenarios.

## Materials and methods

**FIGURE 1:**
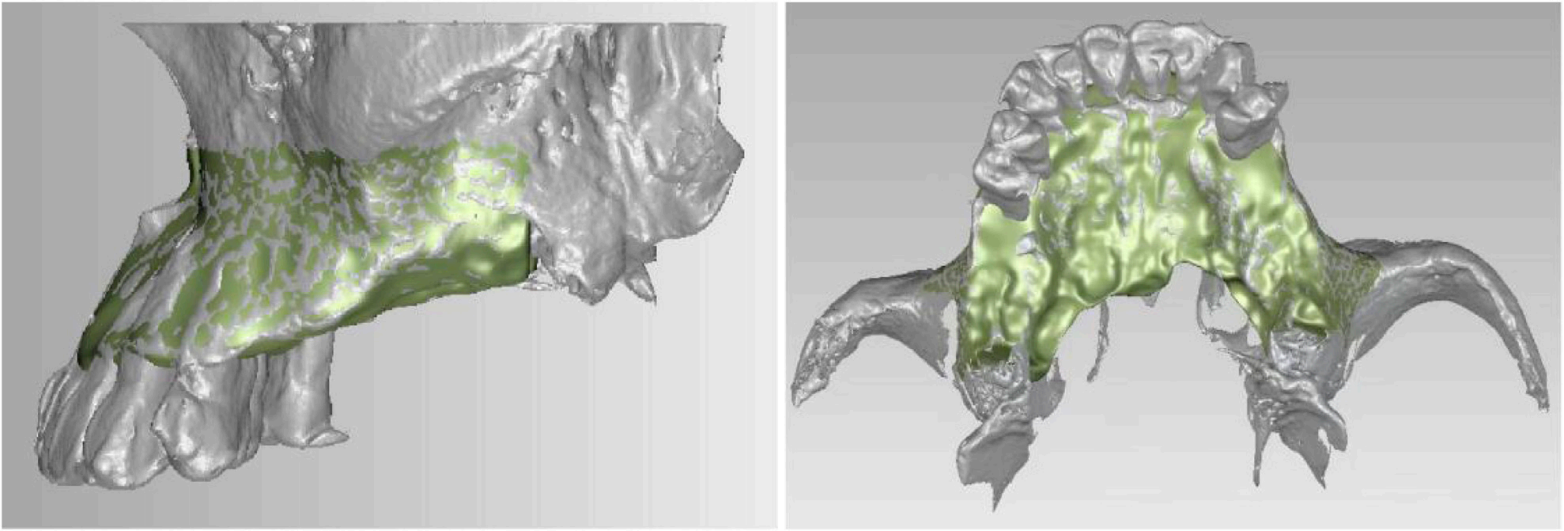
Smoothed maxillary model.

Cone-beam computed-tomography (CBCT) data of a patient was imported to Mimics 21 3D software (Materialise Corporation, Liege, Belgium) for image processing to establish a 3D reconstruction model of the maxilla (Figure 1).

**FIGURE 2:**
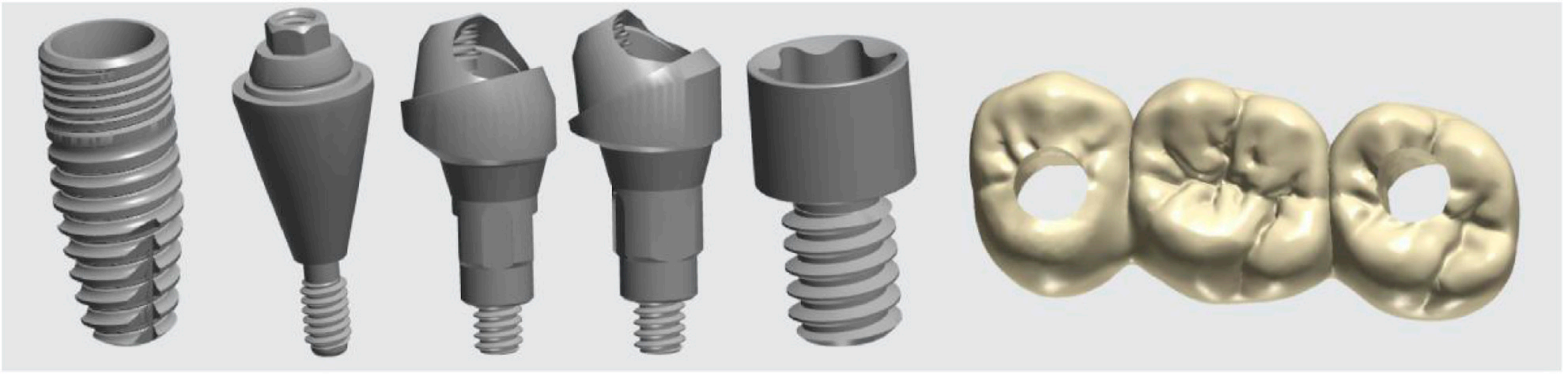
STL data of implant accessories and zirconia bridge.

Stereolithography (STL) data of a dental implant (BLT, 4.1 mm × 10 mm) (Trausim Inc., Changzhou, China), a straight abutment (GH 3.5 mm) (Trausim Inc.), a 17° abutment and its abutment screw (GH 3.5 mm) (Trausim Inc.), a 30° abutment and its abutment screw (GH 3.5 mm) (Trausim Inc.), and prosthetic screws (GH 3.5 mm) (Trausim Inc.) were used designed a three-unit zirconia bridge for the maxillary model by KTJ dental group Co., Ltd (Shenzhen, China) (Figure 2).

**FIGURE 3:**
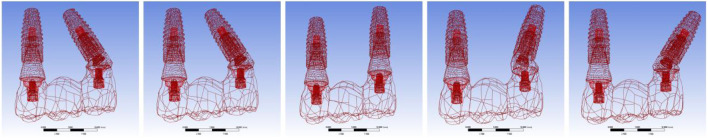
The schematic diagram shows the implant positions in different groups and their mesh distribution of implant, crown, abutment and screw.

The 3D models were imported into the ANSYS Workbench 23.0 software (Ansys Inc., Canonsburg, PA, United States) for generating meshes and defining material properties. We designed five different distal implant implantation directions through assembly, namely, Inner Tilting 30° group, Inner Tilting 17° group, Parallel group, External Tilting 17° group, and External Tilting 30° group. The analysis of the maxilla model was simplified into a cuboid structure with the implant fully placed with a thickness of 6 mm within cancellous bone, with 2 mm thickness of cortical bone on the buccal and lingual side, respectively. The models consist of cortical bone, trabecular bone, implants, abutments, central screws, prosthesis screws, and prostheses. Mechanical window in ANSYS Workbench was used to control the meshing, employing tetrahedral elements. The number of elements and nodes was determined based on the actual model’s characteristics, as an excessive number of elements can increase computational load (Table 1). Minor adjustments was made to the mesh to improve its quality and the accuracy of the subsequent data (Figure 3). The mesh size in this study was 0.7 mm. The material properties of the models were assumed to be isotropic, homogeneous and linearly elastic and all material properties are showed in Table 2. The osseointegration type between the implant and bone tissue is complete osseointegration, with no friction or movement between them; the connection between the implant, crown, abutment, and screw is tight and frictionless, with the contact type set as bonded contact (Tsai et al., 2024; Ayali et al., 2020). The boundary constraints in this study are assumed to be applied to the mesial, distal, and bottom surfaces of the simplified maxillary bone (Tsai et al., 2024; Chen et al., 2024). Such simplifications and setups can speed up the calculations while minimizing their impact on calculation accuracy (Wong et al., 2024; Anitua et al., 2022).

**TABLE 1:** Nodes and elements of each finite element model.

Group	Nodes	Elements
Inner tilting 30°	198,795	115,225
Inner tilting 17°	192,512	111,542
Parallel	189,811	110,546
External tilting 17°	205,012	112,950
External tilting 30°	200,156	115,233

**TABLE 2:** Properties of the materials used in the 3D finite element analysis.

Component	Materials	Young’s modulus (GPa)	Poisson’s ratio v
Dental implant	Titanium	105	0.37
Abutment	Titanium alloy	113.8	0.342
Central screw	Titanium alloy	113.8	0.342
Prosthetic screw	Titanium alloy	113.8	0.342
Three-unit bridge	Zirconium oxide	210	0.3
Cortical bone	Bone	13.7	0.3
Cancellous bone	Bone	1.37	0.3

The maxillary models were submitted to a strict fixation restriction in its upper area. The implants were considered entirely osseointegrated. And the cortical bone was bonded to the trabecular bone. The abutments were fixed in the implants through central screws. Their interfaces considered fixed together. The interfaces between prosthesis and abutments were also considered fixed together. To simulate the occlusal force on the maxilla, two loading types were set in ANSYS Workbench 23.0. A vertical load of 300N acts on the central fovea of each crown occlusal surface, with the force acting perpendicular to the long axis of the tooth. A lateral load of 300N applied on the top/2 of the buccal side of each crown, and the force was 45 angles to the implant.

To comprehend the stress distribution upon loading, von Mises stresses were computed to assess the implant assemblies. Furthermore, the peri-implant bone tissue was scrutinized using ANSYS Workbench 23.0 to monitor the peak stress levels of the models.

## Results

**FIGURE 4:**
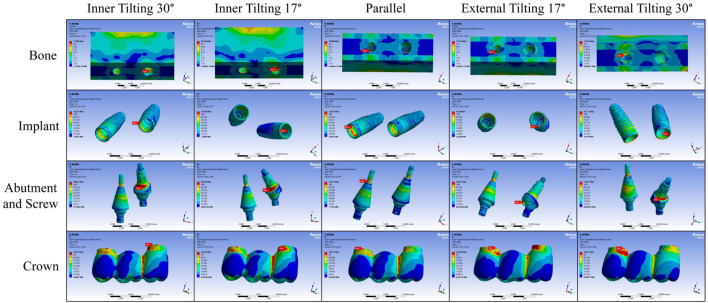
Stress distribution cloud map under vertical force loading.

Analysis of Stress Distribution under Vertical Force Loading. According to the stress distribution cloud map results, for bone tissue, the maximum stress in the inner tilting 17° and 30° groups is located at the lingual side of the distal implant neck corresponding to the bone tissue, while for the parallel group and the inner tilting 17° and 30° groups, the maximum stress is at the mesial implant neck corresponding to the bone tissue. For the implants, only the parallel group exhibits maximum stress at the mesial implant, specifically at the buccal implant neck, while all other groups show maximum stress at the distal implant. Regarding the abutments and screws, the maximum stress for all four tilting groups is also found at the distal abutment. For the inner tilting 17° and 30° groups, it is at the distal abutment neck facing mesially, for the external tilting 17° group, it is at the distal abutment neck facing mesially, and for the external tilting 30° group, it is at the distal abutment neck facing distally. For the parallel group, the maximum stress is distributed at junction of mesial abutment and screw. As for the zirconium oxide bridge, the inner tilting 30° group, the parallel group, and the external tilting 17° and 30° groups all have maximum stress at the buccal side of the junction between distal crown and abutment, but the inner tilting 17° group appears at the mesial crown, slightly above the buccal side of the crown and abutment junction, not at the junction itself (Figure 4).

**FIGURE 5:**
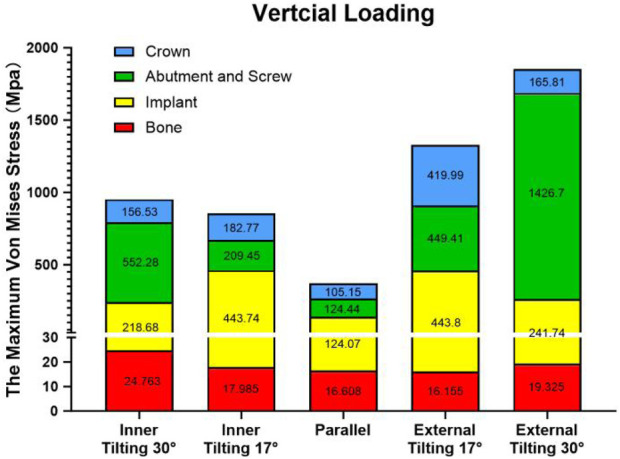
Stacked plots shows the comparison of the maximum stress under vertical force among different groups and various components.

From a detailed data analysis, it is observed that under vertical force loading, the maximum stress experienced by various parts of the parallel group is essentially the smallest among all groups. In the analysis of different tilting groups, we found that regardless of whether it is an inner or external tilting, the greater the angle, the greater the maximum stress borne by the abutment, which may be related to the stress transfer to the implant. Groups with smaller tilting angles bear a greater maximum stress on the implant itself. In the external tilting 30° group, the maximum stress experienced by the abutment reaches as high as 1,426 MPa. However, the maximum stress borne by the abutment in the inner tilting 30° group is not much different from that of the external tilting 17° group, and the maximum stress experienced by the inner tilting 17° group is only 85 MPa higher than that of the parallel group. Overall, the maximum stress values experienced by each subgroup in the inner tilting 17° group are closer to those of the parallel group (Figure 5).

**FIGURE 6:**
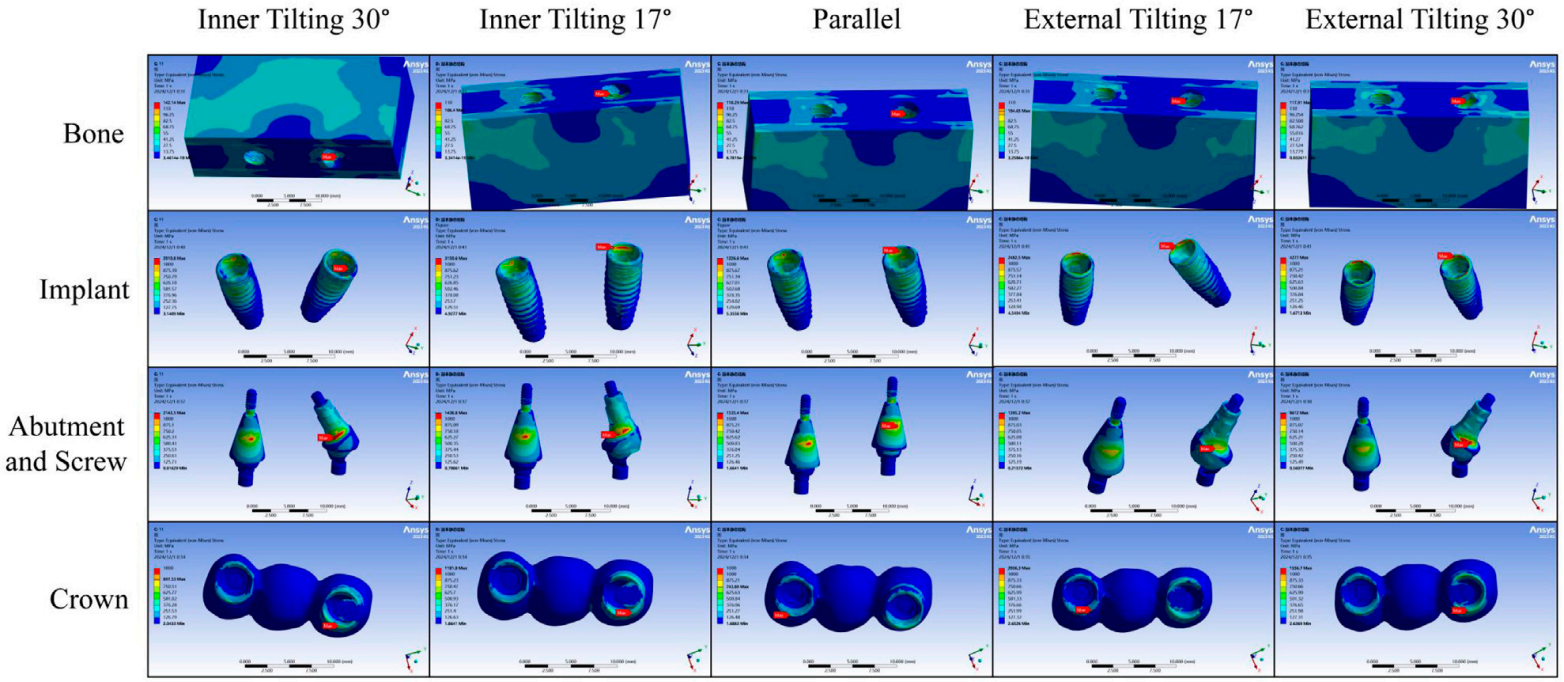
Stress distribution cloud map under lateral force loading.

Analysis of Stress Distribution Cloud Maps under Lateral Force Loading. According to the stress distribution cloud map results, for bone tissue, the maximum stress is located at the bone tissue corresponding to the distal implant neck, except for the inner tilting 30° group, where it is on the lingual side of the distal implant neck corresponding to the bone tissue; all others are on the buccal side of the distal implant neck corresponding to the bone tissue. For the implants, after lateral force loading, the maximum stress appears on the buccal side of the inner wall of the distal implant neck, but for the inner tilting 30° group, it is on the distal side of the inner wall of the distal implant neck. Regarding the abutments and screws, the maximum stress is also found at the distal ones; for the inner tilting 17° and 30° groups, it is at the neck of the distal abutment facing mesially, and for the external tilting 17° and 30° groups, it is at the neck of the distal abutment facing distally, while the parallel group is on the buccal side of the distal abutment. For the zirconium oxide bridge, the inner tilting 17° and 30° groups, as well as the external tilting 30° group, have maximum stress on the buccal side of the junction between the distal crown and abutment, whereas the parallel group and the external tilting 17° group appear on the buccal side of the junction between the mesial crown and abutment (Figure 6).

**FIGURE 7:**
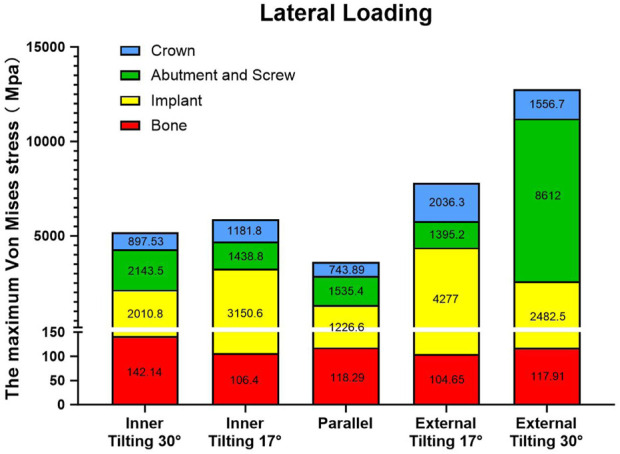
Stacked plots shows the comparison of the maximum stress under lateral force among different groups and various component.

From a detailed data analysis, it is observed that under lateral force loading, the abutment in the external tilting 30° group withstands a maximum stress as high as 8,612 MPa, while the maximum stress endured by the abutments in the other four groups only ranges from 1,300 to 2,200 MPa, significantly lower than that of the external tilting 30° group. The greater the tilting angle, the smaller the maximum stress borne by the implant. The implant in the external tilting 17° group experiences the highest maximum stress, at least 1,000 MPa higher than the other groups. There is little difference in the maximum stress endured by the zirconium oxide bridge and bone tissue across the various groups. Overall, the cumulative maximum stress values borne by each component in the inner tilted 30° group are closer to those of the parallel group (Figure 7).

## Discussion

Due to the presence of the maxillary sinus structure, in this study, we designed five types of maxillary posterior three-unit bridges supported by a mesial axial implant and a distal implant with different insertion angles. Stress distribution and maximum von-mises stress of all implant assemblies including prosthesis, abutment, central screw, prosthetic screw and implant as well as peri-implant bone tissue were computed. Based on the range of bite force for adults during daily eating being 30–300N, taking the upper limit, the load value is therefore set to 300 N (Takaki et al., 2014).

There is no unified standard for the range of von Mises stress within the physiological limits of the Jaw bone (Linetskiy et al., 2017). That is, if the von Mises stress is greater than yield limit stress, then the material is expected to yield (Gong et al., 2016) According to Bayraktar et al., yield strength of trabecular bone tissue is about 82–133 MPa (Bayraktar et al., 2004). The yield strength values are considered as a threshold for microcrack formation in cortical layer of bone. Evidence shows that microcracks stimulate bone remodeling, which begins with bone resorption and is followed by bone formation. It is logical to speculate that a higher frequency of microcrack formation, namely, in shorter intervals will most likely turn the bone response in favor of more resorption (Sadrimanesh et al., 2012). The results of this study showed that the maximum stress value in all groups was between 16 and 25 MPa, which was smaller than the above physiological range. However, during lateral loading, all groups exceeded 100 MPa, but only the inner tilting 30° group exceeded the upper value (133 MPa), indicating that the inner tilting 30° group conducts considerable stress to the bone tissue during lateral loading, which may cause marginal bone resorption. Horizontal forces mainly come from oral parafunctional movements such as clenching or bruxism. Therefore, when applying a design with an tilting of 30° or more, it is necessary to carefully assess the patient’s occlusal condition and it should not be used for patients with excessive bite force. Moreover, whether it is an inner or external tilting, the stress on the bone tissue from a 30° implant tilting is greater than that of 17°. This result is similar to other studies on the stress on bone tissue related to the tilting angle of distal implants in All-on-4 procedures. Their research found that as the tilting degree of the distal implant increases to 15, 30, and 45°, the stress on the bone tissue gradually increases (Sannino, 2015).

Currently, the research results on the biomechanics of distal implant tilting angles are inconsistent, and there is still controversy over the ideal distal implant tilting angle. For distally tilted implants, does different tilting angles affect the stress on the implant? Several scholars have conducted relevant research on this issue. Malhotra et al. conducted a three-dimensional FEA, and the results indicated that there was no significant difference in stress loading between implants with tilt angles of 30° and 40° when the cantilever beam length was consistent (Malhotra et al., 2012). Studies have shown that there is no significant difference in stress loading between implants with front axial placement and implants with distal tilting angles of 15° and 30°, whereas when the tilting angle reaches 45°, stress loading significantly increases, with the greatest pressure at the neck of the implant (Sannino, 2015; Maló et al., 2003). Therefore, based on the relevant studies above, we believe that All-on-4 distal implants will not cause significant negative effects on the peri-implant stress when they are within 30°. However, in our study, we found that the design of three-unit PFDs, whether distal implant is inner tilting or external tilting, whether under vertical or horizontal loading, the maximum stress at the neck of the implant at 17° tilt angle is almost twice that at a 30° tilt angle, which is inconsistent with the results of the All-on-4 study. The abutment and screw are both made of titanium alloy materials, and the yield strength of titanium alloy is about 600–1,600 MPa (Shah and Sivaswamy, 2023; Wong et al., 2023; Wu et al., 2023). When loaded vertically, the maximum stress on the abutment and screw with an 30° external tilt angle reached 1,426.7 MPa, which is close to the upper limit of the yield strength of titanium alloy. When lateral force is applied, the data for internal tilt of 30° and external tilt of 30° exceeded the limit, especially the maximum stress for external tilt of 30° reached 8,612 MPa, which is 6 times more than the external tilt of 17°. Excessive stress can cause plastic deformation or even breakage of the abutment and screw, therefore, the tilt angle of 30° needs to be carefully chosen. This result differs from a study on conventional, V4, M4 all-on-4, which showed that the maximum stress differences for different tilt angles and different abutments were small, all within a range of 1–2 times (Ayali et al., 2020). This may be due to the fact that in the All-on-4 technique, all implants are connected to form an integrated bridge framework, which can distribute stress more evenly due to the splinting effect, whereas this effect is relatively weak in local three-unit bridge restorations. Furthermore, based on the comparison between the implant and the abutment components, we found that when the implant is placed in parallel, the force transmission is relatively even, and the maximum stress values for both the implant and the abutment are basically the same. However, interestingly, we found that at 17°, the maximum stress borne by the implant is the highest among all components, while at 30°, it is the abutment that bears the highest maximum stress. From the distribution results of maximum stress, the cervical areas of the implants and abutments are the main locations of maximum stress distribution. Therefore, for implant manufacturers, local reinforcement is necessary.

Selection of prosthetic material is another critical clinical decision to guarantee even load distribution and longterm implant serviceability. Recently in modern implant dentistry, esthetic requirements, mechanical properties and peri implantitis prevention are considered highly critical perspectives for prosthetic material selection (López Píriz et al., 2019). As a prosthetic material, zirconium oxide with high elastic modulus transmitted less stress to implants and surrounding bone compared to low elastic modulus PEKK, etc. It was reported that the yield strength of zirconia material can reach 1,300–1,500 MPa (Rosentritt et al., 2020). Our results show that during lateral loading, the zirconia bridge restorations in groups external 17° and 30° tilting groups will bear a maximum stress of more than 1,500 MPa, and from the cloud distribution results, the area of the maximum stress occurs at the junction of the near middle crown and the abutment. Adolfi et al. assessed the fracture resistance of two different designs of assembling screw-retained zirconia crowns to titanium bases. In the first design, the titanium bases were cemented to the zirconia crowns using resin cement; in the second design, the zirconia crowns were fixed to titanium bases through a hexagonal connection notched in both the crowns and titanium bases. The authors reported that the group with titanium bases cemented to zirconia crowns had a significantly greater fracture load than the notched restorations. They concluded that the resin cement applied between the restoration and the titanium base could have the potential to improve fracture resistance (Adolfi et al., 2020). Therefore, if the distal implant was externally tilted, the design of full zirconium oxide screw-retained bridge should be avoided.

The study results may, to some extent, provide reference data from a biomechanical perspective for the optimization of implant angulation in patients with continuous loss of three posterior maxillary teeth, helping to reduce the long-term complications associated with the use of implant-fixed bridges. However, This study has several limitations. (1) Although FEA is an effective method, the oversimplification of complex anatomical structures such as the maxilla is a well-known drawback of this type of analysis. (2) This study assumes that the study materials are homogeneous, isotropic, and linearly elastic. (3) The study assumes a 100% implant-bone integration rate, which may not reflect the clinical reality. (4) The current three-dimensional FEA model represents only one patient case. Therefore, these data cannot be considered representative of a sample population. (5) This study only assessed static loads during the chewing process, rather than cyclic loads. Therefore, in practical applications, further clinical research is still needed to verify the feasibility of these results.

Compared to other published materials, this study expands the subject area by focusing on a specific clinical scenario that has not been extensively explored. Previous studies have primarily focused on the biomechanical analysis of implant angulations in edentulous or completely edentulous patients using the All-on-4 technique. Our study, however, investigates the stress distribution in a posterior maxillary region with free-end partial dentition defects (three unit brideg restoration), providing a more detailed understanding of the biomechanical behavior of different implant angulations in this specific context. This research not only fills a gap in the literature but also offers practical implications for clinicians dealing with similar clinical scenarios.

From the results of loading in both vertical and lateral forces, and considering the maximum stress accumulated in all components and bone tissues, it is clear that the parallel group has the least stress. We also found an interesting result that the maximum accumulated stress for the inner tilting group is less than that of the external tilting group. Therefore, from a biomechanical perspective, we believe that if there is a choice between inner or external tilting of the implant, inner tilting implantation is the better option compared to external tilting implantation. Additionally, if only external tilting implantation can be considered, a smaller tilt angle is recommended.

## Data Availability

The original contributions presented in the study are included in the article/supplementary material, further inquiries can be directed to the corresponding author.
